# Transurethral Retrograde Fishing the Double J Urethral Stent: A Tertiary Children Hospital's Experience With a New Technical Strategy

**DOI:** 10.3389/fped.2022.802741

**Published:** 2022-02-25

**Authors:** Chengjun Yu, Chun Wei, Junjun Dong, Xingyue He, Yi Wei, Sheng Wen, Tao Lin, Dawei He, Shengde Wu, Guanghui Wei

**Affiliations:** ^1^Department of Urology, Children's Hospital of Chongqing Medical University, Chongqing, China; ^2^Chongqing Key Laboratory of Children Urogenital Development and Tissue Engineering, Chongqing, China; ^3^National Clinical Research Center for Child Health and Disorders, Chongqing, China; ^4^Chongqing Key Laboratory of Pediatrics Chongqing, Chongqing, China; ^5^Ministry of Education Key Laboratory of Child Development and Disorders, Chongqing, China; ^6^China International Science and Technology Cooperation Base of Child Development and Critical Disorders, Chongqing, China

**Keywords:** double J ureteral stent, transurethral retrograde fishing the double J urethral stent, cystoscopy, hydronephrosis, new technical strategy, ureteropelvic junction obstruction (UPJO), traditional cystoscopic double J urethral stent removal (CJJSR)

## Abstract

**Purpose:**

The purpose of this study is to provide a new strategy for non-cystoscopic double J urethral stent (JJS) removal, the transurethral retrograde fishing the double J urethral stent (TURFJJS), that avoids general anesthesia in pediatric populations.

**Methods:**

We retrospectively reviewed the JJS removal records of patients having ureteropelvic junction obstruction (UPJO). We analyzed differences in the removal success rates, operation-related severe complications, total cost, duration, and parental satisfaction between TURFJJS and traditional cystoscopic double J urethral stent removal (CJJSR) procedures.

**Results:**

A total of 324 patients with UPJO were included in this study. CJJSR yielded a success rate of 100%. TURFJJS achieved a success rate of 94.3%. The TURFJJS was just an outpatient procedure, and its total cost was about 800 Chinese yuan (US$ 124). There were no severe JJS removal-related complications using TURFJJS. Parental satisfaction was 98.2 and 92.5% for the CJJSR and TURFJJS protocols, respectively.

**Conclusion:**

TURFJJS is safe, effective, cost-effective, and well-tolerated in pediatric patients, minimizing or eliminating the need for general anesthesia, additional hospitalization, and waste of time. TURFJJS should be widely used in pediatric urology.

## Introduction

Hydronephrosis is the dilation of the renal collecting system and is one of the most common congenital anomalies in pediatric urology, occurring at a rate of 0.13–0.16% among children (1). The etiology of hydronephrosis is multitudinous with ureteropelvic junction obstruction (UPJO) among the most common causes of childhood hydronephrosis (2). The drainage strategy after pyeloplasty has improved from nephrostomy to ureteral stents, although whether to place an external uretero-pelvic stent (for instance, the Pippi–Salle stent) or an internal uretero-pelvic stent such as double J stent still matters (3–5). Owing to improvements in technology and JJS texture, more recent studies indicate that indwelling of JJS after surgery may yield satisfactory outcomes, with improved user-friendly control, fewer complications, and lower cost (3, 5, 6). A latest network meta-analysis also concluded that compared with other drainage strategies, indwelling of JJS after surgery appears to be more beneficial for pediatric pyeloplasty in view of its ranked results (7).

The JJS should be removed 4–6 weeks after implantation. In adults, the JJS can be removed at an outpatient clinic via traditional cystoscopic double J urethral removal (CJJSR). This operation is safe, quick, and well-tolerated and has a success rate of approximately 100%. However, according to the literature, pediatric patients usually need to be hospitalized, and the removal of the JJS is performed under general anesthesia due to the inability of the patient to tolerate CJJSR under local anesthesia, which greatly increases patient burden and wastes medical resources (8, 9).

A convenient, efficient, and rapid removal of the JJS after adequate drainage, without a requirement for general anesthesia, is urgently needed for pediatric patients. We successfully achieved the first JJS removal using transurethral retrograde fishing of a double J urethral stent (TURFJJS) in December 2016, after a long period of exploration and trial. This new strategy could be conducted in the outpatient clinics, performed in only a few minutes with the help of a common silicone urinary catheter and a prolene suture, and did not require general anesthesia. Using the TURFJJS, we have successfully removed more than 150 JJSs with a success rate of 94.3%. No severe stent removal-related complications have been detected, and the satisfaction level of parents was 92.5%. Here, we introduced this new strategy for JJS removal in pediatrics and reported on our experience.

## Materials and Methods

### Patients Demographics and Definitions

A review of the medical database on JJS removal at Children's Hospital of Chongqing Medical University from January 2012 to September 2020 was conducted. According to the International Classification of Disease 10th version (ICD-10), “N13.3” and “N13.5” were searched in the database first to identify subject-related medical records and then subsequently screened using inclusion and exclusion criteria. Data of cohort patients were extracted based on sex, age, weight, laterality, surgical methods, total cost, duration, JJS removal strategies, JJS removal-related severe complications, and parental satisfaction.

Inclusion criteria were as follows: (1) patients with UPJO who had undergone open or laparoscopic Anderson–Hynes dismembered pyeloplasty and JJS placement during the operation with removal 4–6 weeks after the operation; (2) bilateral UPJO fixed only on one side; (3) no concomitant urogenital abnormalities or systematic mental illness; (4) no urethral stenosis or dilation of the lower ureter or vesicoureteric reflux; (5) complete data information; and (6) no JJS displacement, calculi, prolapse, and obstruction or urinary tract infection (UTI) in the pre-operation evaluation. The exclusion criteria were as follows: (1) hydronephrosis with ureteral polyp, calculi, distortion, or horseshoe kidney, hypospadias and other urogenital deformities; (2) urethral catheter or nephrostomy used for drainage after the operation; (3) postoperative indwelling or removal <4 weeks after the operation; (4) patients with secondary UPJO or having a history of ureteral surgery; and (5) incomplete data.

All patients with indwelling JJS received oral nitrofurantoin at a dosage of 1 mg/kg per day to prevent UTI after surgery, with JJS removal performed between 4 and 6 weeks. A routine urinalysis and kidney–ureter–bladder (KUB) radiography were arranged prior to JJS removal to exclude UTI, JJS displacement, prolapse, and calculi requiring hospitalization and CJJSR. To facilitate screening before JJS removal and rapid clinical judgment, UTI was defined as the presence of clinical symptoms and/or microscopic observation of urinary sedimentation with a white blood cell count above 5 under a high magnification field. Parents were told to have a routine urinalysis in the outpatient department or local hospital 1 week after JSS removal with a phone call follow-up for UTI and recording of parental satisfaction. Parental satisfaction was divided into “satisfied,” “neutral,” and “unsatisfied,” and the reason for dissatisfaction was noted. We successfully conducted the first JJS removal *via* TURFJJS in December 2016. From that time, rigorously selected participants were treated by TURFJJS and provided with CJJSR if the former failed. Participants were defined as those of age >6 months and <12 years old and without UTI, JJS displacement, prolapse, or calculi.

Written informed consent was obtained from the legal guardians. The hospital ethics committee approved this study.

### Surgical Technique

#### CJJSR

After satisfactory general anesthesia and routine disinfection, the cystoscope was inserted, and the bladder wall and trigone of the bladder were examined. The JJS was located and the foreign body forceps used to clamp the JJS under direct observation. It was then removed, and the patient was examined for bleeding, before completing the operation.

#### TURFJJS

Materials: Articles for disinfection and hole towel, 1% povidone iodine, silicone catheter of appropriate size (F6 or F8), needle prolene suture (4–0), and lidocaine gel paste.

The child is quieted, placed in a supine position in bed, and cleansed with disinfectant using lay hole towels.

Lidocaine gel paste is applied to the outer opening of the urethra and kept for 1–2 min.

A needle prolene suture is passed through the silicone urethral catheter and inserted into the bottom of the bladder (usually, there will be a resistance when inserted catheter reached the bottom).

The urethral catheter is stabilized at the outer opening of the urethra, and the prolene suture pulled outward to form a bow at the inner end of the silicone urethral catheter.

The suture and catheter are secured at the outer opening of the urethra and rotated clockwise or counterclockwise for four to six cycles until resistance is felt, indicating that the catheter and suture have wrapped and wound around the JJS. If no resistance is felt, then this step is repeated.

The catheter and suture are stabilized, and then, the catheter and JJS are drawn out of the body.

Disinfectant is applied and the procedure completed. No oral or intravenous analgesia is needed.

**Figure 1:**
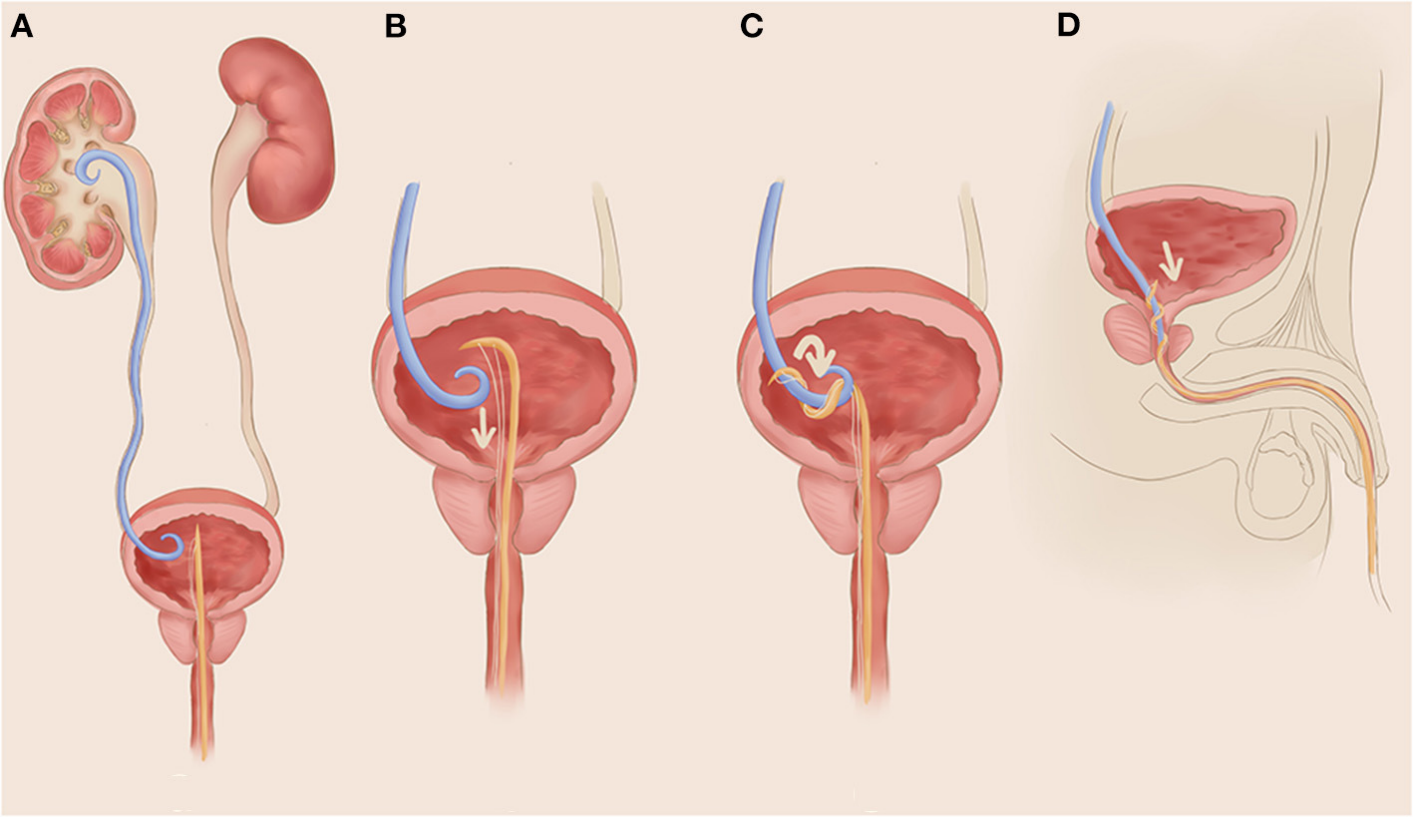
A simplified schematic diagram of transurethral retrograde fishing the double J urethral stent (TURFJJS). **(A)** A needle prolene suture pass through a conventional silicone urethral catheter and is inserted into the bottom of the bladder; **(B)** stabilize the urethral catheter at the outer opening of the urethra, and pull outward the prolene suture to form a bow of the inner end part of the silicone urethral catheter; **(C)** fix the suture and catheter at the outer opening of the urethra and rotate them clockwise or counterclockwise for four to six cycles until obvious resistance is felt, indicating that the catheter and suture have wrapped and enwinded around the double J urethral stent (JJS); **(D)** stabilize the catheter and suture and gently pull out the catheter with appropriate tension, together with the JJS out of the body.

A simplified schematic diagram of TURFJJS is shown in Figure 1, and a demonstration video is also provided in the Supplementary Material.

The patient was observed postoperatively for 2 h and was allowed to leave following normal urination with a reminder for a 1-week follow-up. All TURFJJS was carried out by three senior attending doctors.

### Outcome Parameters

The JJS removal success rate, transfer rate, total cost, duration time, removal-related severe complications, UTI after operation, and parent satisfaction were compared to evaluate the safety, efficacy, tolerability, and application of TURFJJS (Table 1).

**Table 1:** A summary of sociodemographic characteristics and outcome parameters between transurethral retrograde fishing the double J urethral stent (TURFJJS) and traditional cystoscopic double J urethral stent removal (CJJSR).

**Characteristics**	**Participants, No. (%)**		***P*-value^**$$**^**
	**Group A^**##**^(*n* = 150)**	**Group B^**##**^ (*n* = 165)**	
**General information**			
**Sex**			
Male	124 (82.7)	133 (80.7)	0.637
Female	26 (17.3)	32 (19.3)	
Age, mean (SD), m	48.5 (42.5)	91 (89)	NA^%%^
Weight, mean (SD), kg	19.25 (5.75)	19.75 (10.25)	NA^%%^
Laterality^**^			
Left	109 (72.7)	116 (70.3)	0.643
Right	41 (27.3)	49 (29.7)	
**Operation**			
Laparoscopic	131 (87.3)	136 (82.4)	0.226
Open	19 (12.7)	29 (17.6)	
**JJS removal assessment**			
Success rate	94.3% (150/159)	100%	-
Transfer rate	5.7% (9/159)	0	-
Total cost, CNY(USD), mean (SD)	About 800/124	3278/508 (120/19)	-
Total duration, mean (SD), d	0.5	2.5 (0.5)	-
**JJS removal-related severe complications**			
Gross hematuria	1	0	-
Urethral rupture	0	0	-
Transient dysuria	1	0	-
Urinary tract infection	3	2	-
Satisfaction rate of parents	92.5%^ΓΓ^ (147/159)	98.2% (162/165)	-

*JJS indicates double J urethral stent; SD, standard deviation; TURFJJS, transurethral retrograde fishing the double J urethral stent; CJJSR, traditional cystoscopic double J urethral stent removal; UTI, urinary tract infection; UPJO, ureteropelvic junction obstruction; CNY, Chinese yuan; USD, united states dollar*.

## *Group A, TURFJJS, 159 attempted and 9 transferred to CJJSR; Group B, CJJSR, 9 cases transferred from TURFJJS not included for comparison*.

$$ *The dichotomous variables was tested by χ2 test; the continuous variables did not conform to the normal distribution, and was tested by the independent sample Mann-Whitney U test*.

%% *Group A participants were highly selected, age >6 month and <12 years old. Group A and B was not baseline comparable in age and weight*.

** *For bilateral UPJO patient, only one side operated included, and then allocated to the corresponding side*.

ΓΓ *Reason for “Neutral” or “unsatisfied” was: failed after several attempts, hematuria, uncontrolled crying or do not cooperate for attempts, parents refused for another attempt after the first-time TURFJJS*.

The success rate was defined as removal of the JJS without severe complications, such as gross hematuria, urethral injury, or rupture. A failed TURFJJS was defined as the unsuccessful removal of the JJS after three to five attempts, parental refusal to have another try after the first attempt, or severe complications. UTI after operation was defined as children needing antibiotic treatment as judged by the outpatient department and dysuria with an obvious filling of the bladder, and disposable catheterization was needed after sufficient induction.

### Statistical Methods

All statistical analyses were performed using the SPSS 25.0 software. Paired variables were presented as ratios (%) and compared by χ^2^ test. Continuous variables were presented by mean ± standard deviation (SD) and analyzed by the Shapiro–Wilk test. The *t*-test was used if it conformed to a normal distribution, and if not, the independent sample Mann–Whitney U test was applied. A *p* < 0.05 was considered statistically significant.

## Results

**Figure 2:**
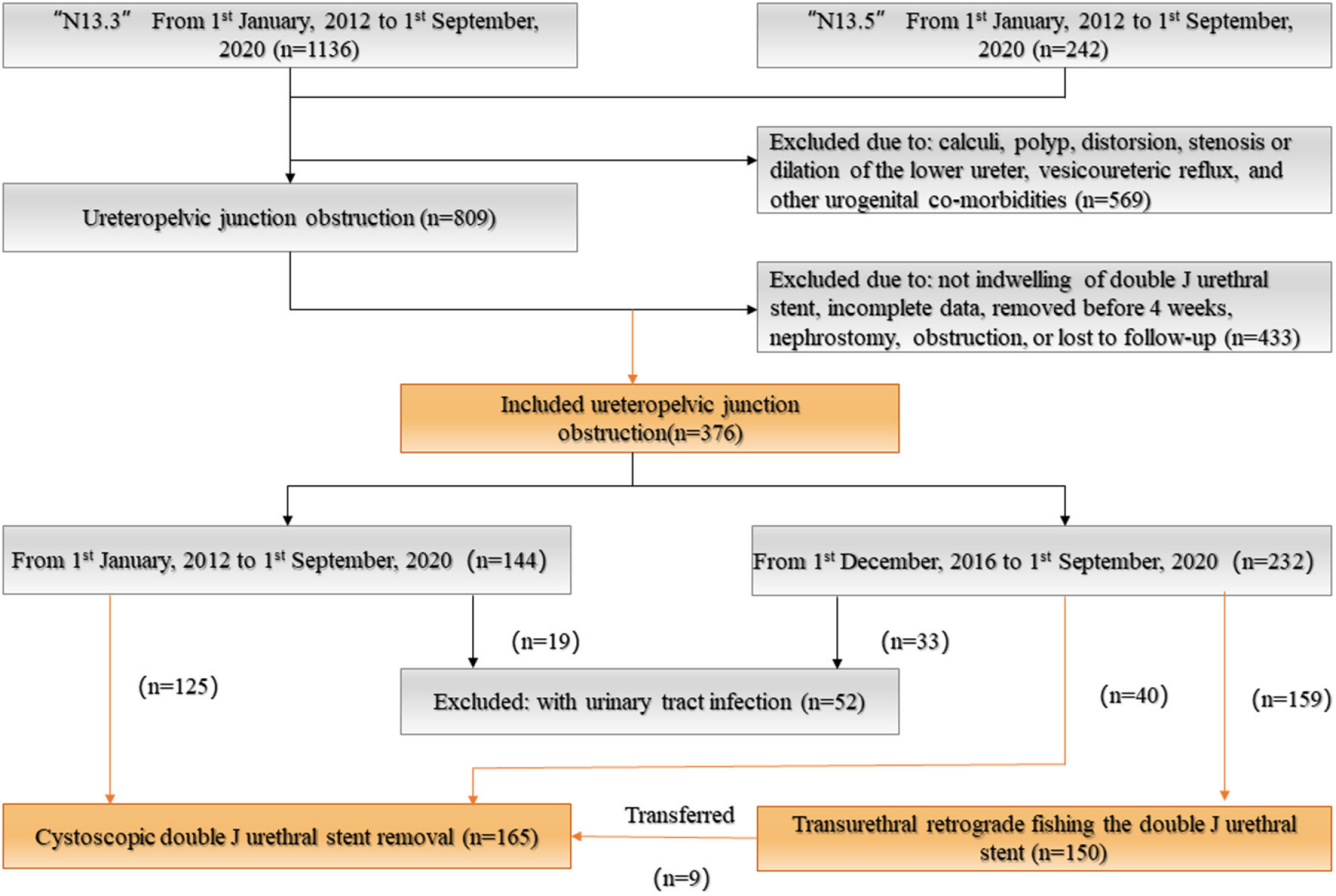
Flow diagram of case inclusion, exclusion, and technical methods for double J ureteral stent removal.

A total of 324 patients with UPJO were included in the final study. Of these cases, 165 underwent CJJSR, whereas 159 cases were treated by TURFJJS. A detailed flow diagram showing case inclusion, exclusion, and technical methods is presented in Figure 2. One hundred and fifty cases were successfully extubated via TURFJJS (group A), and 165 UPJO cases undergoing CJJSR (group B). No statistical difference between group A and group B were observed with respect to sex, laterality, and operation methods (*P* > 0.05). Age and weight baselines were independent for group A–selected participants (Table 1).

The CJJSR success rate was 100%. The TURFJJS method had a success rate of 94.3%, of which nine cases having removal failures were transferred and successfully completed using the CJJSR method. The cases information and possible reasons for these nine children who transferred to CJJSR are shown in Table 2. The average total cost was 3278.03 ± 120.01 CNY (508/19 USD), and the hospital duration in group B was 2.5 ± 0.5 days. The total cost for group A was about 800 CNY (124 USD), and the duration was about 0.5 days, from outpatient clinical evaluation to the return home, both much less than CJJSR. In addition, the TURFJJS only took a few minutes to fish the stent. There were no severe removal-related complications in group B. In the TURFJJS group, one case displayed hematuria that spontaneously relieved and one case had transient dysuria needing catheterization in which urethral rupture did not occur. The number of cases that needed additional oral antibiotic intervention during the first postoperative week in groups A and B was 3 and 2, respectively, and no cases required hospitalization. Parental satisfaction percentages in groups A and B were 92.5 and 98.2%, respectively. The reasons for dissatisfaction in TURFJJS were as follows: failure after three to five attempts with transferal to CJJSR (*n* = 4), uncontrolled crying or lack of cooperation for any attempts (*n* = 2), hematuria (*n* = 1), parents' refusal to undertake another attempt after the first attempt (*n* = 3), or unknown (*n* = 2) (Table 1). The reasons for dissatisfaction with CJJSR was the occurrence of a refractory UTI and high cost (*n* = 1), long hospital stay (4 days, because of the weekends, *n* = 1), and unknown (*n* = 1).

**Table 2:** Cases information and possible reasons for these 9 children who transferred to traditional cystoscopy stent removal from TURFJJS (Ordered by operation time).

**Cases**	**Sex**	**Age, m**	**Weight, kg**	**Laterality**	**Pyeloplasty**	**Operation time**	**KUB**	**The possible explanation**
#1	Male	23	14.5	Left	Open	9 December, 2016	Figure 3B	JJS lower straight, paralleled anterior-posterior body
#2	Male	12	8	Left	Laparoscopic	31, December, 2016	Figure 3C	JJS reached right bladder wall cross the trigone of bladder from the left
#3	Male	6	7.5	Right	Open	1 Feburary, 2017	Not special	Unknown, maybe small age
#4	Male	35	18	Left	Laparoscopic	16, March, 2017	Figure 3D	JJS folded in the trigone of bladder
#5	Male	53	20.5	Left	Laparoscopic	20, September, 2017	Not special	Uncontrolled crying, do not cooperate
#6	Male	33	17	Left	Laparoscopic	15, March, 2018	Figure 3E	The coil of lower JJS was much more broad than usual, and high folded in the bottom of bladder
#7	Male	84	26.5	Right	Open	12, December, 2018	Not special	Uncontrolled crying, do not cooperate
#8	Male	140	42	Left	Laparoscopic	17, August, 2019	Figure 3F	Bladder capacity was large, and the coil of lower JJS broadened a lot
#9	Female	14	12	Right	Laparoscopic	4 January, 2020	Not special	JJS slipped from the prolene suture and urethral catheter and left in the urethra

## Discussion

To avoid cystoscopy for removal of the JJS, Kajbafzadeh et al. attempted to connect a feeding tube to the JJS, which was secured to the external body skin via a separate stab incision made during pyeloplasty and was removed along with the feeding tube after 3–4 weeks in 2014 (10). This strategy provided for the possibility of non-cystoscopic stent removal and elimination of urethral catheterization, although some conditions like perirenal skin infection, urinary leakage, difficulties during nursing, and errors in the pulling out of the traction feeding tube still need to be improved. In 2020, Issi et al. reported a new method for JJS removal without anesthesia, which was performed in 14 children who had undergone a ureteroneocystostomy operation (11). In their method, the JJS was tied to the urethral catheter by a suture and retrieved postoperatively on the fourth day without cystoscopy and anesthesia. The length of JJS indwelling was not long enough, and this procedure increased discomfort.

Our new strategy solves the problem of extra hospitalization for JJS removal under general anesthesia in pediatric populations. Compared with two newly reported non-cystoscopic removal methods (10, 11), TURFJJS allowed for a longer period of postoperative drainage as it did not require early removal of the JJS, and TURFJJS did not require any externalization of catheters and thus avoided the possibility of urinary leakage around any externalized catheter and avoided the embarrassed situation that a drainage tube would be pulled out by accident after surgery. TURFJJS employed the natural cavity, was minimally invasive, had a high success rate and satisfaction, and could be done in outpatient clinics. Hence, it should be widely used in pediatric urology.

After 4 years of exploration and summary, the success rate of TURFJJS has steadily improved in our department. The main elements of this process are summarized as follows.

Relaxation and cooperation: Relaxing young patients contributes much to the whole process by decreasing complications, especially in older children. Doctors and parents should pacify the child to calm the patient or at least to prevent resistance. In some cases, analgesia or sedation could also be considered.

Local anesthesia: Satisfactory local infiltration of lidocaine into the external urethral lessens the stimulation of the urethral mucosa and is beneficial for relieving fear and improving the JJS removal success.

Forming a “bow” from the inner end part of the silicone urethral catheter: After insertion of the catheter and prolene suture, the urethral catheter at the outer opening of the urethra is stabilized and pulled outward along the prolene suture to form a bow of the inner end part of the silicone urethral catheter, increasing the chance to unwind the JJS.

Stabilizing the catheter and suture under tension after unwinding and pulling the JJS out slightly: When rotating the catheter and suture to wrap and unwind the JJS, the suture should be maintained tightly together with the catheter. As when applying tension to the JJS, JJS slippage should be avoided and maintained in the urethra.

Attention to the child's reactions: It is wise to stop if the child experiences extreme pain or if hematuria is observed.

During the operation, excessive force should not be used nor rotation to avoid ureteral injury or rupture, especially when pulling the JJS out is strongly resisted. In addition, transfer to CJJSR should be considered to determine what may be blocking removing of JJS after three to five attempts of TURFJJS.

**Figure 3:**
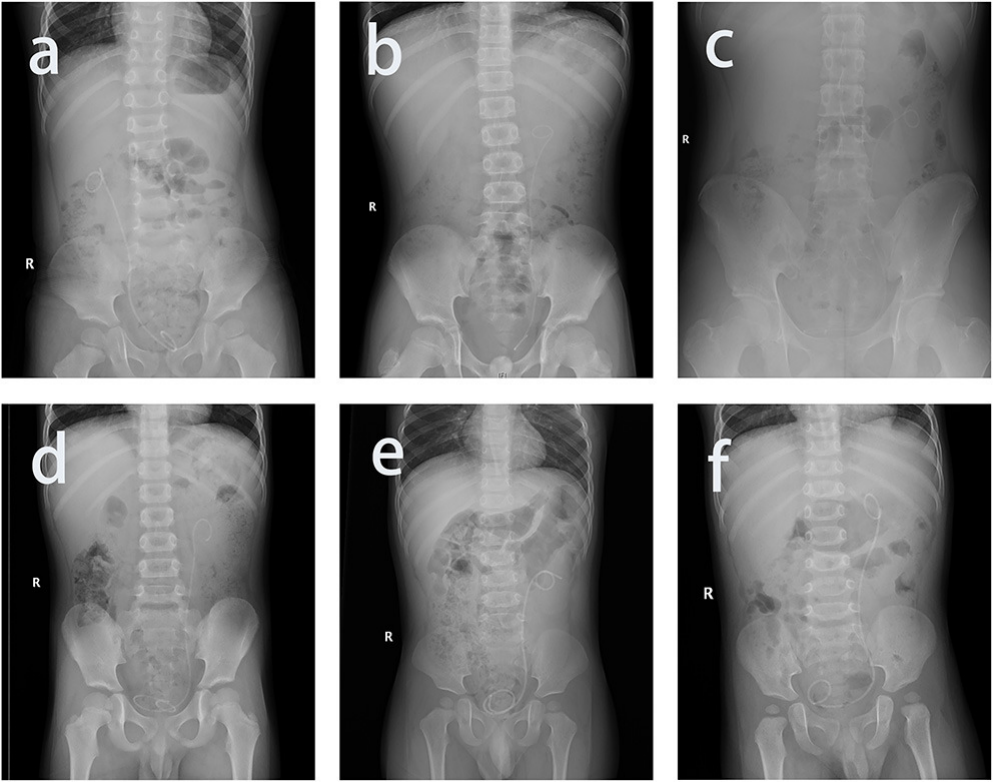
The possible reason for failed transurethral retrograde fishing the double J urethral stent in kidney–ureter–bladder radiography manifestation. **(a)** The ideal model with satisfactory position, the lower double J urethral stent (JJS) bent into a coil, and the direction of the coil crosses the anterior–posterior body in some way; **(b)** lower JJS been almost straight, paralleled anterior–posterior body; **(c)** JJS reached right bladder wall cross the trigone of bladder from the left; **(d)** JJS folded in the trigone of bladder; **(e)** the coil of lower JJS was much more broad than usual and high folded in the bottom of bladder; **(f)** bladder capacity was large, and the coil of lower JJS broadened a lot.

The most commonly used size of silicon catheter was F6 and F8. Usually, F6 was used for patients <4 years, and F8 was used for patients aged 4–12 years. The most commonly used size of prolene suture was 4-0. Gradually, the type of urethral catheter and the size of suture are not strict restrictions any more, and even a balloon Foley catheter also works. Smaller-sized catheters may decrease discomfort if they do not affect the forming of the “bow” and wrapping the JJS. Conclusions from failures of the TURFJJS process: Likely reasons for failure of TURFJJS in KUB manifestation are as follows: a straight lower end of the JJS, JJS proximity to the bladder wall, parallel anterior–posterior body, elevated positioning, a folded JJS, and large bladder capacity (Figure 3). In these cases, consultants from guardians regarding TURFJJS failure should be obtained or directly perform the CJJSR. As a consequence, pre-operative KUB evaluation is important in skilled practice. Apart from UPJO, we have already applied the new procedure in ureteral JJS removal or pyelolithotomy, ureteroneocystotomy, and other urogenital operations. Limitations due to young age are gradually being overcome.

No extra drainage, urethral catheter, JJS, or nephrostomy after dismembered pyeloplasty in UPJO remains controversial in current studies, although JJS and nephrostomy are widely used in urology (3–5, 12). Because an externalized nephrostomy is exposed for long intervals, bacteria may enter the renal pelvic through the external tube, resulting in a risk of infection. Moreover, the operation is complicated, and the exposed catheter falls off easily, disturbing the child (13). Furthermore, indwelling JJS is not easy to misdischarge, which is more suitable for pediatric population (14). Currently, the main disadvantage of an indwelling inner JJS is the difficulty of extra hospitalization that is needed for JJS removal under general anesthesia (4). TURFJJS is a new remedy method for this condition, and we pointed out that its widespread use will greatly increase the use of internal JJS in pediatric urology.

The TURFJJS procedure, nonetheless, has limitations. TURFJJS cannot guarantee 100% JJS removal rate, and application conditions require strict control. In addition, we have not mastered all the key points enabling successful first-time attempts at JJS removal. In addition, the pre-operative evaluation cannot be quantified or programmed yet. We are still exploring quantification of the technical parameters and improving the TURFJJS technique with regard to patients' characteristics, KUB evaluations, and indwelling JJS status. At last, patients' experience and pain were not quantitatively evaluated in this study. An improvement for future prospective study would be to survey patients' satisfaction from older children and to evaluate the level of pain/discomfort.

## Conclusion

In a word, although limitations exist, TURFJJS is safe, effective, cost-effective, and well-tolerated in pediatric patients, minimizing or eliminating the need for general anesthesia, additional hospitalization, and waste of time. TURFJJS should be widely used in pediatric urology.

## Data Availability Statement

The original contributions presented in the study are included in the article/Supplementary Materials, further inquiries can be directed to the corresponding author.

## Ethics Statement

The studies involving human participants were reviewed and approved by Children's Hospital of Chongqing Medical University. Written informed consent to participate in this study was provided by the participants' legal guardian/next of kin.

## Author Contributions

CY, SWe, and SWu: study conception and design. CY and CW: data acquisition. CW, JD, and XH: analysis and data interpretation. CY and YW: drafting of the manuscript. DH, TL, SWu, and GW: critical revision. All authors approved the final version of this manuscript.

## Funding

This work was supported by National Natural Science Foundation of China (No. 81873828), Chongqing Municipal Health Commission (High-Level Medical Reserved Personnel Training Project of Chongqing), Innovation Program for Chongqing's Overseas Returnees (cx2019030), and the Senior Medical Talents Program of Chongqing for Young and Middle-aged.

## Conflict of Interest

The authors declare that the research was conducted in the absence of any commercial or financial relationships that could be construed as a potential conflict of interest.

## Publisher's Note

All claims expressed in this article are solely those of the authors and do not necessarily represent those of their affiliated organizations, or those of the publisher, the editors and the reviewers. Any product that may be evaluated in this article, or claim that may be made by its manufacturer, is not guaranteed or endorsed by the publisher.

## Abbreviations

JJS: double J ureteral stent
UPJO: ureteropelvic junction obstruction
KUB: kidney–ureter–bladder radiography
TURFJJS: transurethral retrograde fishing the double J urethral stent
CJJSR: traditional cystoscopic double J urethral stent removal
UTI: urinary tract infection.
